# Pharmacodynamic assays to facilitate preclinical and clinical development of pre-mRNA splicing modulatory drug candidates

**DOI:** 10.1002/prp2.158

**Published:** 2015-06-26

**Authors:** Yihui Shi, Amanda S Joyner, William Shadrick, Gustavo Palacios, Chandraiah Lagisetti, Philip M Potter, Lidia C Sambucetti, Stefan Stamm, Thomas R Webb

**Affiliations:** 1 Division of Biosciences, SRI InternationalMenlo Park, California, 94025; 2 Department of Chemical Biology and Therapeutics, St. Jude Children’s Research HospitalMemphis, Tennessee, 38105; 3 Department of Molecular and Cellular Biochemistry, University of Kentucky741 South Limestone, Lexington, Kentucky, 40536

**Keywords:** Cancer, exon-skipping reporter, in vivo imaging, pre-mRNA splicing, spliceosome modulators, sudemycin D6

## Abstract

The spliceosome has recently emerged as a new target for cancer chemotherapy and novel antitumor spliceosome targeted agents are under development. Here, we describe two types of novel pharmacodynamic assays that facilitate drug discovery and development of this intriguing class of innovative therapeutics; the first assay is useful for preclinical optimization of small-molecule agents that target the SF3B1 spliceosomal protein in animals, the second assay is an ex vivo validated, gel-based assay for the measurement of drug exposure in human leukocytes. The first assay utilizes a highly specific bioluminescent splicing reporter, based on the skipping of exons 4–11 of a Luc-MDM2 construct, which specifically yields active luciferase when treated with small-molecule spliceosome modulators. We demonstrate that this reporter can be used to monitor alternative splicing in whole cells in vitro. We describe here that cell lines carrying the reporter can be used in vivo for the efficient pharmacodynamic analysis of agents during drug optimization and development. We also demonstrate dose- and time-dependent on-target activity of sudemycin D6 (SD6), which leads to dramatic tumor regression. The second assay relies on the treatment of freshly drawn human blood with SD6 ex vivo treatment. Changes in alternative splicing are determined by RT-PCR using genes previously identified in in vitro experiments. The Luc-MDM2 alternative splicing bioluminescent reporter and the splicing changes observed in human leukocytes should allow for the more facile translation of novel splicing modulators into clinical application.

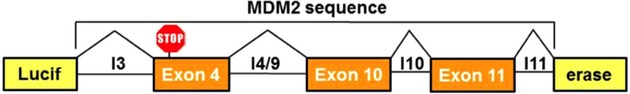



## Introduction

Nearly all polymerase II transcripts undergo pre-mRNA splicing, which is the joining of exons and the removal of introns to form mature mRNA. The spliceosome, a large multiprotein/RNA complex, catalyzes pre-mRNA splicing. The spliceosome is composed of at least 170 proteins and five snRNAs (small nuclear RNAs) (Behzadnia et al. 2007). The snRNAs are associated with proteins forming the U1, U2, U4, U5, and U6 snRNPs.

**Figure 1:**
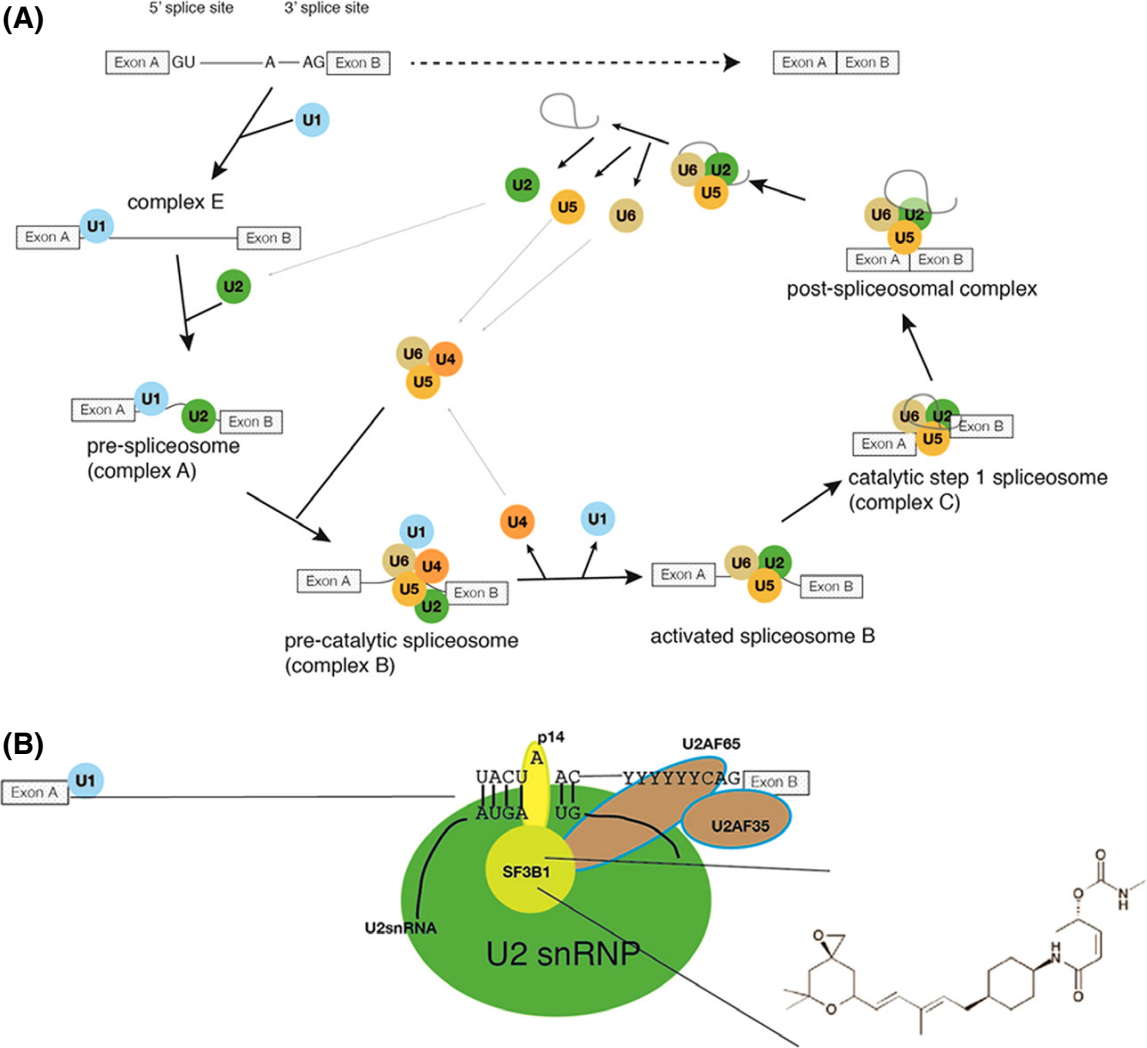
Schematic overview of the splicing reaction. (A) Exons are shown as boxes, introns as lines. The spliceosome recognizes the 5′ and 3′ splice sites and the branchpoint (indicted by the “A” in the intron). Splicing results in the joining of the exons and removal of the intron (dotted arrow). Splicing starts with the early (E) complex formation that contains U1 snRNP recognizing the 5′ splice site through RNA:RNA interaction. The entry of U2 snRNP marks the formation of complex A that forms complex B after the entry of U4/U6/U5 snRNPs. The B complex is activated through the exit of U4 and U1, leading to rearrangements of the U2/U5/U6 snRNPs that allows catalysis in complex C. The catalysis results in the joining of exons and the release of the former intron as a lariat. After the splicing reaction, these snRNPs dissociate from the postspliceosomal complex and the lariat is degraded. (B) Recognition of the branchpoint sequence through U2 snRNP in the A complex. U2 snRNA binds the RNA surrounding the branchpoint adenosine and leads to a “bulging out” of the adenosine, which is contacted by the U2 component p14. In addition, the U2 component SF3B1 contacts the U2AF protein, located at the 3′ splice site. In this arrangement, the 5′ splice site, the branchpoint and the 3′ splice site are recognized by the spliceosome. Sudemycin D6 is a promising cancer drug that binds to SF3B1, the structure is shown as an insert.

Exons are defined by the 5′ splice site, the 3′ splice site, and the branch point. The spliceosome recognizes these elements and then assembles, in a stepwise manner, onto the nascent pre-mRNA (see Fig.1). First, the U1 snRNP binds to the 5′ splice site thereby forming the “early (E) complex.” This is then followed by the binding of splicing factor 1 (SF1) to the branch point, which in turn facilitates the binding of the U2AF factor (U2 auxiliary factor) on the 3′ splice site. Upon the stabilization of U2 snRNP binding, SF1 is displaced by the SF3 complex, which results in an interaction between U2AF65 and SF3B1 that are components of the U2AF and SF3B complexes, respectively. The U2 snRNP participates in an RNA:RNA interaction with the branch point, which leads to the recognition of the branchpoint adenosine. Through the exchange and recruitment of other factors, the “A complex” is transformed into the spliceosomal “B complex” that removes an intron and joins the exons by a trans-esterification reaction. The intron then undergoes debranching and is subsequently degraded (Kramer 1996).

Most pre-mRNAs contain exons that can be alternatively spliced, that is, they can be either removed as part of introns or included in a mature mRNA. Given the importance of alternative splicing for normal cellular function, it is not surprising that aberrant regulation of alternative splicing can lead to human disease. This connection to human health is increasingly recognized and has been covered in numerous reviews (Wang et al. 2007; Kim et al. 2008; Tazi et al. 2009, 2010; Barta and Schumperli 2010; Dhir and Buratti 2010; Hallegger et al. 2010; Raponi and Baralle 2010; Younis et al. 2010). In most cases, changes in alternative splicing are caused by point mutations, which have been curated in databases (Bechtel et al. 2008a,b).

It is notable that deregulation of alternative splicing is also recognized as a hallmark of cancer (Klinck et al. 2008; Venables et al. 2009; Misquitta-Ali et al. 2011; reviewed in Kim et al. 2008; David and Manley 2010). Recently strong evidence has accumulated that aberrant splicing of pre-mRNA is a driver of tumorigenesis and that modulation of this process may be a promising novel target for cancer therapy (Bonnal et al. 2012; Webb et al. 2013). Groundbreaking discoveries in this area include the identification of recurrent mutations in the SF3B1 splicing protein in numerous cancers including myelodysplastic syndromes (Graubert et al. 2012), chronic lymphocytic leukemia (Rossi et al. 2011; Damm et al. 2012; Quesada et al. 2012), acute myeloid leukemia (Murati et al. 2012), breast cancer (Cancer Genome Atlas 2012), lung adenosarcoma (Imielinski et al. 2012), and uveal melanoma (Furney et al. 2013). The selective sensitivity of tumors to agents that target SF3B1 has also been linked to overexpression of MYC (Hubert et al. 2013).

Prior to the identification of recurrent mutations of SF3B1 in cancer, two independent groups published landmark reports demonstrating that the antitumor natural products FR901464 (Kaida et al. 2007) (FR) and pladienolide (Kanada et al. 2007) both target SF3B1. Thus, progress in natural product screening, target identification, medicinal chemistry applied to spliceosome modulators, and high-throughput transcriptome sequencing has led to a remarkable convergence of independent research that identified a new oncology drug target and a new class of potential small-molecule therapeutic agents. Subsequently we reported the design and synthesis of FR analogs that contain only three chiral centers (the sudemycins, including C1, D1, sudemycin D6 [SD6], and F1) (Fan et al. 2011; Lagisetti et al. 2013), and focused our work on the lead-optimization of the sudemycins, based on FR structure-activity relationships and a hypothetical consensus pharmacophore model (Lagisetti et al. 2008). This work ultimately led to SD6, which is currently in preclinical development (Lagisetti et al. 2013). In the following we describe two types of novel pharmacodynamic assays that facilitate drug discovery and development of this intriguing class of innovative therapeutics; the first assay is useful for preclinical optimization of small-molecule agents that target the SF3B1 spliceosomal protein in animals, the second assay is an ex vivo validated, gel-based assay for the measurement of drug exposure in human leukocytes. This assay is a starting point for the development of a pharmacodynamic assay that will require future clinical validation in combination with pharmacokinetic data for patients that are undergoing treatment with a drug such as SD6.

## Materials and Methods

### Methods

#### Cell culture and drug treatment

SK-MEL-2 cells were purchased from American Type Culture Collection and cultured in minimum essential medium with Earle’s salts and l-glutamine containing 1 mmol/L sodium pyruvate, 10% fetal bovine serum (FCS) and 10 mmol/L HEPES. The pediatric rhabdomyosarcoma Rh18 cells were kindly provided by Dr. P. Houghton (Ohio State University – Nationwide Children’s Hospital, Columbus, OH). Rh18 cells were cultured in RPMI 1640 medium containing 10% FCS and 10 mmol/L Hepes. Cells are all maintained in humidified incubator with 5% CO_2_ at 37°C. Sudemycins and herboxidiene were synthesized in the Webb lab. Pladienolide B was purchased from EMD Millipore Corp (Billerica, MA, U.S.A.). Compounds were dissolved in DMSO at 10 mmol/L as stock solution. DMSO concentration was kept constant at 0.5% in all drug treatments.

#### Reporter construction

Plasmid containing MDM2 minigene sequence was obtained from Dr. D. Chandler (Nationwide Children’s Hospital) (Singh et al. 2009). The Luc-MDM2 construct contains an MDM2 minigene sequence within the luciferase ORF in the pGL4.51 vector (Promega, Madison, WI, U.S.A.), which contains synthetic firefly luciferase luc2 gene under the control of CMV promoter. Intron 3 through Intron 11 of the MDM2 minigene sequence is inserted at nucleotide 477 of the luciferase ORF. A stop codon was engineered into exon 4 of MDM2.

#### Transient transfection and generation of stable cell lines

Single transfection of RL-CMV (Promega) or co-transfection of Luc-MDM2 and RL-CMV in SK-MEL-2 cells with or without sudemycin D1 treatment was performed as follows: On day 1, 2.5 × 10^6^ SK-MEL-2 cells were seeded into a 10 cm petri dish with 10 mL media and incubated for 24 h at 37°C. On day 2, cells were transfected with RL-CMV plasmid either alone or together with the Luc-MDM2 construct using TransIT-2020 reagent (Mirus Bio LLC, Madison, WI, U.S.A.) and incubated for 24 h at 37°C. On day 3, cells were harvested and plated into a 96-well plate (10^4^ cells/well) and incubated for 24 h at 37°C. On day 4, cells were treated with indicated sudemycin D1 concentrations and incubated for 4 h at 37°C. Firefly luciferase activity was measured after the addition of the Dual-Glo^**®**^ reagent (Promega), subsequently Renilla luciferase activity was measured after adding Stop & Glo^**®**^ reagent (Promega). All luciferase activity measurements were performed on an Envision plate reader (Perkin Elmer, Waltham, MA, U.S.A.).

To establish a stable cell line expressing Luc-MDM2, the SK-MEL-2 cells were first transfected with the Luc-MDM2 plasmid and subjected to selection by changing the media daily in the presence of G-418 (1 mg/mL) for 2 weeks. The pooled stable cells were trypsinized, counted, and seeded sparsely (5 cells in one 100 mm petri dish) to assure that each colony would be formed from a monoclonal population. We selected 30 colonies and characterized each viable clone by measuring its responsiveness to SD6 treatment. Briefly, cells were plated at a density of 10^4^ cells/well in 96-well plates (Greiner, Bio-One, Monroe, NC, U.S.A.) for overnight culture. The following day, cells were treated with serial dilutions of SD6 for 4 h and One-Glo (Promega) reagents were added to measure the luciferase activity using an Envision plate reader. Clone YS2.5 was selected for subcloning due to its robust growth and very large dynamic range in response to SD6 treatment. The clones and subclones were periodically frozen down at early passages and retained for future work.

#### Isolation of human lymphocytes

The blood draws were approved by the internal review board at University of Kentucky. Blood donors underwent overnight fasting to reduce lipids in the blood, which facilitated later RNA isolation. The blood was added to platelet storage bags (Blood storage, Inc., Seattle, WA, U.S.A.) and incubated with 1 *μ*mol/L SD6 or 1 *μ*mol/L DMSO as a control for up to 12 h. Lymphocytes are collected in a Ficoll gradient using Histopaque columns (Sigma-Aldrich, St. Louis, MO, U.S.A.) after 0–12 h of treatment. RNA was extracted from the lymphocytes using Trizol (Invitrogen, Carlsbad, CA, U.S.A.). The alternative splicing of different target genes was analyzed by RT-PCR, as previously described (Convertini et al. 2014).

#### RT-PCR assay

Following exposure of either Rh18 cells or isolated human lymphocytes to drugs for determined periods of time, total RNA was prepared using an RNeasy Mini kit (Qiagen, Valencia, CA, U.S.A.). After conversion to cDNA using High capacity RNA-to-cDNA kit (Life Technologies, Waltham, MA, U.S.A.), PCR was performed using specific oligonucleotides using standard protocols. Products were analyzed using agarose gel electrophoresis and ethidium bromide staining. The intensities of the PCR bands were quantified using gel-densitometry. The *t*-test was used to determine the significance of the results.

Primers to test splicing changes in Rh18 cells: MDM2_FOR: CTGGGGAGTCTTGAGGGACC; MDM2_REV: CAGGTTGTCTAAATTCCTAGGG; Ubiquitin A_FOR:ACCTGACCAGCAGCGTCTGATATT; Ubiquitin A_REV: TCGCAGTTGTATTTCTGGGCAAGC.

Primers to test splicing changes in human lymphocytes: RPp30_FOR:GAGGCCTGGCTTTTGAACTT; RPp30_REV:CCTTGGCGTCACTTTCAGAG; DUSP11_FOR:GACATCAAGTGCCTGATGATGA; DUSP11_REV:ATGTCCCCGGCACCTATT; SRRM1_FOR:GACTCTGGCTCCTCCTCCTC; SRRM1_REV:GGACTTCTCCTCCGTCTACCA; PAPOLG_FOR:AAGAGATCCCATTCCCCATC; PAPOLG_REV:TGCGTGATGTATCAATAGTTGGA; *β*-ACTIN_FOR:AGAGCTACGAGCTGCCTGAC; *β*-ACTIN_REV:GGATGTCCACGTCACACTTC.

#### Subcutaneous xenograft model, bioluminescence imaging, and histology

SRI International’s IACUC approved all animal studies. Female nude mice were obtained from Taconic (Hudson, NY, U.S.A.) and were 6–8 weeks of age at initiation of experiments. Five million SK-MEL-2/Luc-MDM2 cells in 100 *μ*L of 1:1 mixture of phosphate buffered saline (PBS) and matrigel (BD Biosciences, Franklin Lakes, NJ, U.S.A.) were subcutaneously inoculated in the flank of each mouse under 2% isoflurane anesthesia. After the tumors had grown to approximately 50–80 mm^3^ in volume, the animals were randomized into groups (3 mice per group) according to their basal bioluminescence signal and their tumor volume. Bioluminescence signal was obtained by intraperitoneal injection of d-luciferin (200X luciferin stock solution, 30 mg/mL in PBS, dose of 150 mg/kg) for 20 min. Bioluminescence imaging was performed with IVIS spectrum (Perkin Elmer) under constant isoflurane (2%) anesthesia. The acquired bioluminescence signals in a region of interest (ROI) over the tumor site were recorded as the total flux radiance (p/sec/cm^3^/sr) with Living Image software (Perkin Elmer). SD6 was formulated in vehicle, 10% hydroxypropyl-beta-cyclodextrin (HPCD) in phosphate buffer (pH 7.4) in a stock concentration of 1.5 mg/mL. Different doses of SD6 were administered intratumorally at single doses alongside of the vehicle controls once a day for 5 days. Mice were killed and the tumors were excised and weighed at 21 days following the final drug and vehicle treatment. Tumor tissues were fixed in 10% neutral-buffered formalin, processed for paraffin embedding, sectioned and hematoxylin and eosin (H&E) stained. Sections were examined under a microscope (Leica DM5000B, Wetzlar, Germany) equipped with a CCD video camera and photographed.

## Results

### Novel luc-MDM2 Construct and the exon-skipping luciferase reporter assay

**Figure 2:**
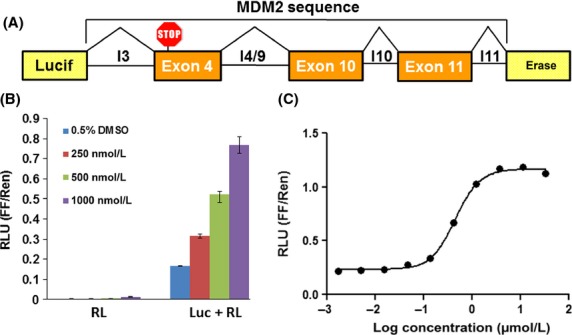
Characterization of the exon-skipping reporter. (A) The Luc-MDM2 construct contains parts of an MDM2 minigene sequence (intronic and exonic sequences) within the luciferase ORF in the pGL4.51 vector. (B) SK-MEL-2 cells transfected with the RL-CMV plasmid alone (RL) or Luc-MDM2 and RL-CMV plasmids (Luc + RL) and treated with either 0.5% DMSO or different concentrations of sudemycin D1 for 4 h. Dose response curve of sudemycin D1 in Luc-MDM2/RL-CMV dual reporter assay. SK-MEL-2 cells were co-transfected with Luc-MDM2 and RL-CMV plasmids and treated with indicated sudemycin D1 concentrations for 4 h. RLU, relative luminescence units; FF/Ren, firefly/Renilla

In order to facilitate the development of spliceosome modulators we sought to design a reporter assay to rapidly report the exon-skipping activity in the context of an intact cell. We selected the proto-oncogene MDM2 as the basis for our reporter because its alternative splicing in cancer is well characterized and we have previously reported a PCR-based assay that is highly specific for inhibitors of SF3B1 (Fan et al. 2011). MDM2 is an E3 ubiquitin ligase and chief negative regulator of the tumor suppressor protein p53 and it is encoded by 12 exons (Wu et al. 1993; Bartel et al. 2002). A splice variant, containing only exons 3 and 12, leads to the stabilization of p53, resulting in cell cycle arrest and/or apoptosis (Evans et al. 2001). Our previous studies on MDM2 minigenes (Singh et al. 2009) revealed that treatment with sudemycins induces alternative splicing such that exons 4–11 of MDM2 are skipped (Fan et al. 2011). On the basis of these observations, we designed an exon-skipping Luc-MDM2 reporter (Fig.2A); that requires skipping MDM2 exons 4, 10, and 11, inserted into the firefly luciferase (Luc) gene, to produce full-length luciferase transcript and bioluminescence.

We selected SK-MEL-2 cells for transfection with the Luc-MDM2 reporter, based on this cell line’s high sensitivity to SD6 (Lagisetti et al. 2013). We co-transfected a constitutive Renilla expression vector (RL-CMV) into SK-MEL-2 to normalize the Luc signal for transfection efficiency. Co-transfection of the Luc-MDM2 with RL-CMV in SK-MEL-2 cells produced low-luminescent background signals in the absence of sudemycin, as the 5′ end of the luciferase sequence was predominantly spliced to MDM2 exon 4, which contains a translational stop codon (Fig.2A). In the presence of sudemycin a 5-fold increase in luminescence was detected compared to the vehicle control, which is both time and dose dependent (Fig.2B and C).

### Development of a stable SK-MEL-2/Luc-MDM2 cell line

**Figure 3:**
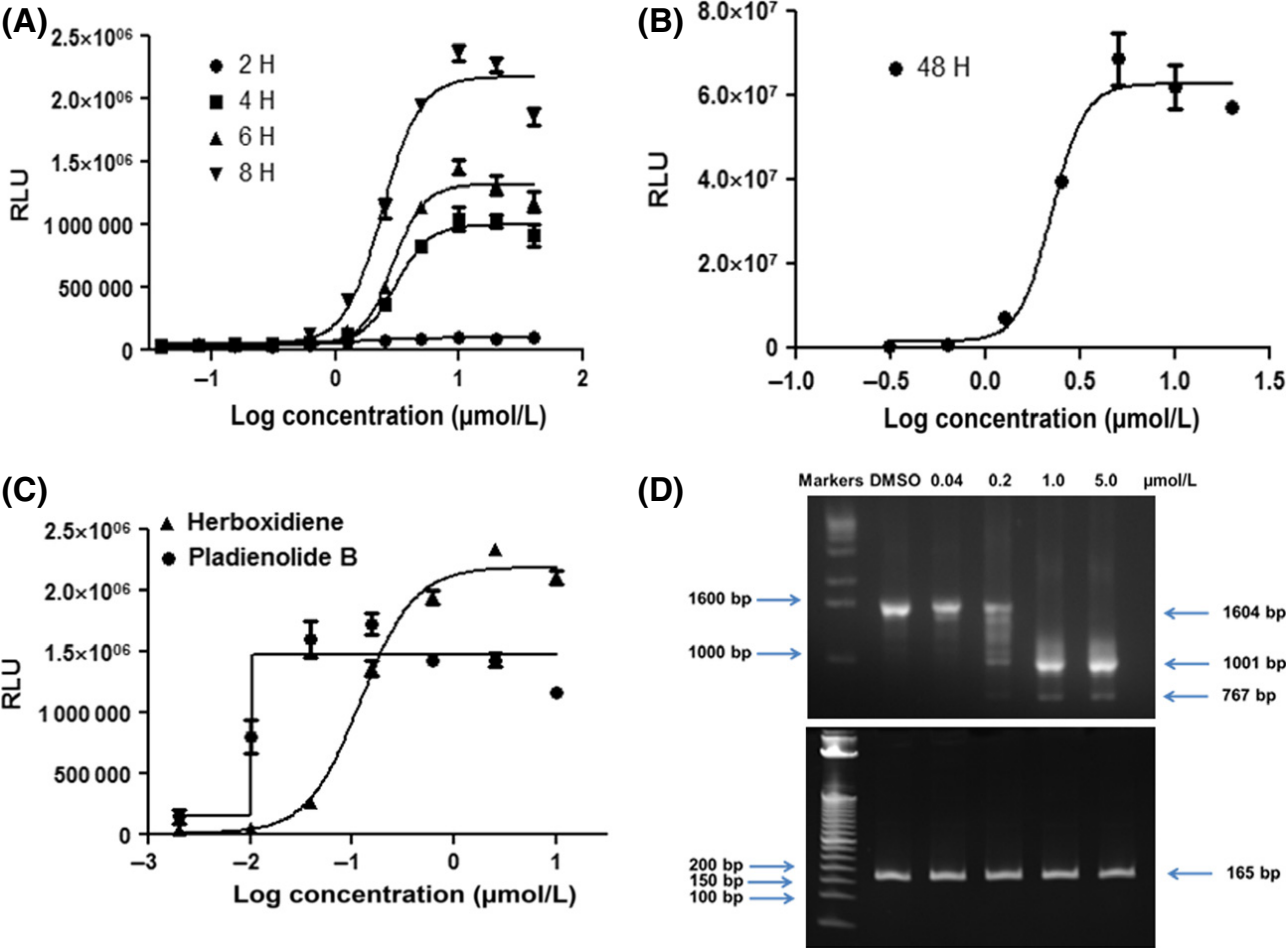
SD6 Dose response, orthogonal RT-PCR assay, and assay validation. (A, B, and C) SK-MEL-2/Luc-MDM2 stable cells were plated at a density of 5000/well in 96-well plates and incubated overnight at 37°C in 5% CO_2_. The following day, cells were treated with serial dilutions of SD6 for 2, 4, 6, 8 (A), 48 (B) hours, or Herboxidiene or Pladienolide B (C) for 4 h, ONE-Glo reagents (Promega) were added to measure the luciferase activity. (D) RT-PCR was performed in Rh18 cells. PCR products (in bp) are indicated by the arrows; 1604 bp represents the full-length MDM2 transcript. 1001 and 767 bp represents alternative MDM2 splicing. Ubiquitin transcripts (165 bp) were used as a control.

In order to develop an in vivo pharmacodynamic assay with broad applications we established an SK-MEL-2 cell line stably expressing the same Luc-MDM2 reporter (SK-MEL-2/Luc-MDM2) as described above. No co-expression of RL-CMV was needed in this model as the cells were stably transfected. We conducted dose-response and time-course assessments of this novel stable reporter cell line and observed increasing amounts of luciferase activity with increasing concentrations of SD6 and exposure time in vitro. Strong luciferase activity was obtained with 4–8 h incubation time (Fig.3A); however, incubation times up to 48 h produced even higher luciferase activity (500-fold increase from baseline at 48 h, Fig.3B). Incubation times of 4 h or more produced large dynamic ranges (about 30-fold increase from baseline) in vitro, such that 4 h was chosen as the time point for further in vitro assays. Maximum luciferase activity was obtained at approximately 10 *μ*mol/L at every time point. Furthermore, we confirmed the potent exon-skipping activity of two other SF3B1 targeted agents (pladienolide B and herboxidiene) (Kotake et al. 2007; Hasegawa et al. 2011; Lagisetti et al. 2014) in this stable cell line (Fig.3C).

As noted above we have previously reported an orthogonal RT-PCR assay for exon skipping, which can be used to validate hits from the Luc-MDM2 screen (Fan et al. 2011). In this RT-PCR assay the maximum effects were noted at 1–5 *μ*mol/L (Fig.3D).

### In vivo bioluminescent pharmacodynamic (PD) assays

**Figure 4:**
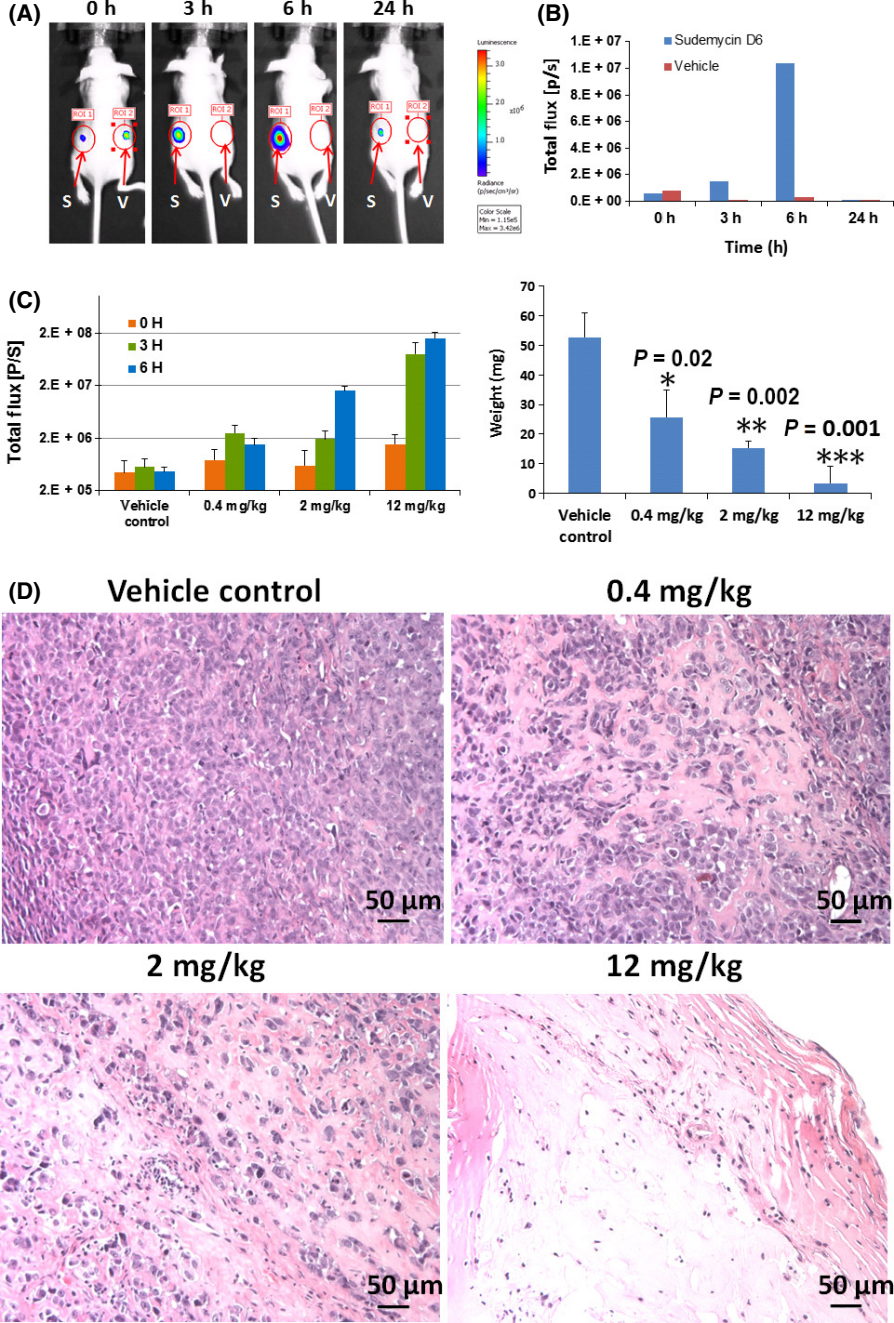
In vivo measurement of spliceosome modulation by bioluminescent imaging BLI. (A) Athymic (nude) mice were injected s.c. with SK-MEL-2/Luc-MDM2 cells in both left and right flanks. Representative pictures of one animal treatment at different time points are shown. Either vehicle control (V) on the left frank or 12 mg/kg SD6 (S) on the right flank were intratumorally injected into mice. Mice were imaged at 0, 3, 6, and 24 h after injection. (B) Regions of interest (ROIs – red circles) were quantified with ImageQuant software. (C) SK-MEL-2/Luc-MDM2 cells were injected s.c. in right flanks of nude mice. Vehicle control or three dose levels of SD6 were injected intratumorally into established tumors. Mice were imaged 0, 3, and 6 h after injection of SD6 and ROIs were quantified, and dosed once daily for four more consecutive days. After 3 weeks, mice were sacrificed, tumors excised and weighed. *P*-values determined by Student’s *t-*test versus vehicle control are shown. * p = 0.02, ** p = 0.002, *** p = 0.001 (D) H&E stained sections of tumor tissues. Images were taken at 200× magnification.

We recognized an intriguing advantage of the SK-MEL-2/Luc-MDM2 stable reporter cell line in that it can be used for in vivo bioluminescent imaging (BLI) to rapidly and specifically assess the in vivo PD of splicing modulatory drugs. In conjunction with administration of the luciferase substrate d-luciferin, this enables real time non-invasive measurement of alternative splicing in an animal model. We report here that the SK-MEL-2/Luc-MDM2 stable cell line can form subcutaneous (s.c.) tumors in immunocompromised mice, and that these tumors respond to SD6, as measured by induction of the luciferase reporter compared to a vehicle control (Fig.4A). Using this model we established that an intratumoral injection of 12 mg/kg SD6 produced a marked increase in Luc activity, with 32-fold induction of signal observed 6 h post dosing (Fig.4B). We further observed increasing luciferase activity with increasing doses of SD6 by intratumoral injection (Fig.4C). No adverse effects were noted on the behavior or health of the animals during the study.

We evaluated the effects of repeated intratumoral SD6 administration on the tumor xenografts, by dosing once daily for 5 days. We observed that the induction of luciferase activity decreased on the second day and after (data not shown). We postulated that the decrease in luciferase activity after the first dose might be due to a decrease in the viability of the tumor cells. In order to investigate the relationship between intratumoral SD6 administration and tumor viability we allowed tumors to grow for three additional weeks following the final SD6 administration and measured the weight of the tumors. Notably, tumor weights were significantly reduced by intratumoral treatment with SD6 in a dose-dependent manner. Remarkably, all three SD6 treated tumors completely regressed at the highest dose. Histological analysis revealed a high degree of necrosis at 2 mg/kg, some necrosis at 0.4 mg/kg and predominantly viable tumor cells in the vehicle control (Fig.4D). We are now using the SK-MEL-2/Luc-MDM2 stable reporter cell line to efficiently optimize formulations and schedules of SD6 by i.v. administration.

### Potential human pharmacodynamic biomarkers

In anticipation of future clinical trials we are also pursuing the development of pharmacodynamic splicing biomarkers. In previous studies, we identified a panel of genes that change alternative splicing patterns in Rh18, HeLa and HEK293 cells after the treatment of sudemycin E, a close analog of SD6 (Convertini et al. 2014). In this study, we initiated the ex vivo treatment of human blood samples with SD6 from volunteers using fresh samples for SD6 treatment, under standardized conditions. The effect of SD6 ex vivo in fresh blood samples directly reports the change in the alternative splicing patterns in primary human cells following drug exposure.

**Figure 5:**
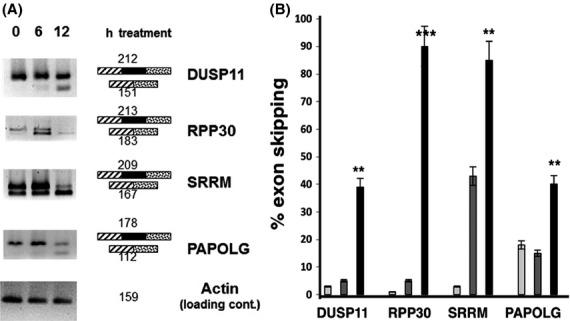
Alternative splicing changes from sudemycin treatment of human blood. (A) Freshly drawn human blood samples were treated with 1 *μ*mol/L SD6 and incubated for 0–12 h. The splicing patterns were analyzed by end-point RT-PCR, as previously described (Convertini et al. 2014). The structure of the RT-PCR products is indicated schematically next to the gels. Numbers indicate the sizes of the RT-PCR products. (B) Quantification of splicing changes in four human subjects. The shading of each bar indicates the time of drug treatment; Off-white: 0 h; Gray: 6 h; Black: 12 h. The error bars indicate standard errors between individuals, **P < 0.01, ***P < 0.001.

As seen in Figure5, we found that DUSP11, RPp30, SRRM1, and PAPOLG genes were highly expressed in primary human lymphocytes and exhibited alternative splicing changes after SD6 treatment. Exon-skipping was found in all four genes at both 6-h and 12-h time points (Fig.5A). We further quantified the percentage of exon skipping of those genes in treated blood samples of four human subjects and found that the alternative splicing changes were statistically significant with *P*-values <0.01 or <0.001 (Fig.5B).

## Discussion

A general picture of the role of SF3B1 as an important regulator of pre-mRNA 3′-splice site fidelity has emerged (Corrionero et al. 2011). The binding of U2 snRNP to the pre-mRNA branch site is one of the most critical steps in establishing the catalytic center of the spliceosome. During this binding process, an RNA-RNA duplex is formed between the branch point sequence in the intron and the branch point-binding region located in the U2 RNA of the U2 snRNP. The SF3 protein complex interacts with U2AF65, a protein that facilitates recognition of the 3′ splice site (see Fig.1). It has been shown that the natural product FR targets SF3B1 and acts by preventing binding of the U2 snRNA to the pre-mRNA, thereby altering the conformation of the branch point sequence and resulting in the generation of alternatively spliced mRNAs (Corrionero et al. 2011; Folco et al. 2011). In addition, agents that target SF3B1, such as the sudemycins, have been shown to alter splicing patterns and induce exon skipping in genes such as MDM2 (Fan et al. 2011; Lagisetti et al. 2013). The proposal that the spliceosome is a valid target for cancer (Bonnal et al. 2012; Webb et al. 2013) has been strongly supported by recent work (Maguire et al. 2015). Additionally, the oncogenic function of SF3B1 mutations, which leads to the selection of cryptic 3′ -splice sites, has very recently been elucidated (DeBoever et al. 2015). It has been observed that the cancers that are associated with recurrent spliceosome mutations are uniformly heterozygous, implying that some nonmutant and fully functional spliceosome is required for cell viability. These observations allow for the development of a hypothetical mechanism that can also account for the observed exquisite sensitivity of certain tumors to SF3B1 targeted agents, whereas normal cells are relatively resistant to drug (Lagisetti et al. 2013; Webb et al. 2013), as cancer cells with this type of aberrant splicing are likely sensitive to the complete knockdown of SF3B1 activity.

In order to discover drugs that target splicing we have previously reported a sensitive and specific low-throughput cell-based assay for alternate splicing of MDM2 in the Rh18 cell line (Fan et al. 2011). We now describe the development of a high-throughput, cell-based luciferase reporter with multiple applications in spliceosome modulator drug discovery and development. There have been reports of efficient screening for modulators of alternate pre-mRNA splicing (Arslan et al. 2013). Early studies of the natural product splicing modulators mostly focused on intron retention as an important readout of splicing “inhibition” (Kaida et al. 2007; Kotake et al. 2007; Younis et al. 2010). However, subsequent groundbreaking work showed that dramatic changes in exon-skipping are induced by spliceostatin A and that the loss of the fidelity of the 3′-splice site selection is the fundamental change induced by this natural product derivative (Corrionero et al. 2011). Our exon-skipping reporter system has the distinct advantage of being able to identify splicing modulatory agents that are relevant to cancer biology and would be missed using intron retention-based splicing activity assays (Younis et al. 2010). We postulate that specific alternatively spliced transcripts in exon-skipping could be identified and serve as predictive biomarkers for splicing modulator drug action in ongoing preclinical as well as in future clinical studies.

In addition, this reporter enables efficient evaluation of targeted activity in relevant in vivo models and will expedite identification of novel spliceosome modulators and accelerate preclinical development of anticancer agents such as SD6. Additionally, in anticipation of future clinical trials and in recognition of individual-specific alternative splicing, we have successfully investigated the use of blood-based biomarkers that will enable the assessment of splicing modulatory drug exposure in humans based on alternate splicing changes that we have discovered in drug treated human tumor cells (Convertini et al. 2014). These animal and human pharmacodynamic assays have broad application in drug discovery and development for splicing modulatory antitumor drugs.

Spliceosome targeted drug development has been hindered by the dearth of suitable targeted screening assays. The cell-based exon-skipping luciferase reporter described here may also have the potential to identify novel spliceosome modulators in vitro and also serve to bridge into late-stage in vivo lead compound optimization. We are currently in the process of testing the potential of this construct as a screening tool and will report the results of this work in the future. Thus, the Luc-MDM2 cell line provides two unique tools for both drug discovery and development of novel splicing modulator drug leads.

BLI has been demonstrated to be a valuable technique for evaluation of drug efficacy (Vassileva et al. 2008) and functional activity in noninvasive animal models (Weber et al. 2013). Here, we extended the use of BLI to monitor mechanism-based activity for pharmacodynamic studies and for our future studies of formulation, scheduling, and optimization that are needed for drug development. The novel application of this bioluminescence-based model offers a high signal-to-noise ratio, resulting in a highly sensitive assay for targeted activity of splicing modulators in real time in vivo. In addition, drug combinations of splicing modulators with other targeted drugs can be explored to determine optimal doses and the sequence of administration of drugs like SD6 in order to maximize targeted efficacy in vivo.

Separately we have developed an ex vivo assay to measure the drug exposure in human leukocytes using genes that may serve as a starting point in the development of pharmacodynamic biomarkers for future clinical studies. The four genes that we have identified in this study can be analyzed in order to quantify the percentage of exon skipping to serve as markers that can be tested in a clinical setting using drug pharmacokinetic data. If validated this type of pharmacodynamic marker may be useful from the preclinical phase through all of the clinical phases of drug development. In the future, we will correlate the confirmed alternative splicing of these markers to drug dose levels, to the time after administration and to the circulating drug levels, with a significant panel of patients receiving SD6 in a clinical setting. Further validation and completion of the pharmacodynamic biomarker identification will help guide future clinical trials, as those biomarkers can be adapted for use in screening patient samples, and could be used to show drug effects in blood over the time coarse of the treatment. Assays of this type will allow for the development of a more optimal dosing regimen and schedule thus improving patient benefit and the quality of future clinical trials.

## Author Contributions

Webb, Sambucetti, Stamm, Potter, Joyner, and Shi participated in research design. Shi, Joyner, Shadrick, Palacios, and Lagisetti conducted the experiments of this study. Shi, Joyner, Shadrick, Stamm, Sambucetti, and Webb performed data analysis. Shi, Sambucetti, Stamm, and Webb contributed to the writing of the manuscript.

## Disclosures

The authors Webb and Lagisetti disclose that they are inventors on a US patent and a patent application related to sudemycin and associated technology. This patent is assigned to St. Jude Children Research Hospital.

## Abbreviations

BLI: bioluminescent imaging
Luc-MDM2: Luciferase construct containing MDM2 intron and exons
SD6: Sudemycin D6
